# *Cuscuta campestris* induces apoptosis by increasing reactive oxygen species generation in human leukemic cells

**Published:** 2018

**Authors:** Maliheh Moradzadeh, Azar Hosseini, Hasan Rakhshandeh, Azita Aghaei, Hamid Reza Sadeghnia

**Affiliations:** 1 *Golestan Rheumatology Research Center, Golestan University of Medical Sciences, Gorgan, Iran*; 2 *Pharmacological Research Center of Medicinal Plants, Mashhad University of Medical Sciences, Mashhad, Iran*; 3 *Division of Neurocognitive Sciences, Psychiatry and Behavioral Sciences Research Center, Mashhad University of Medical Sciences, Mashhad, Iran*

**Keywords:** Cuscuta campestris, Leukemia, Apoptosis, Differentiation, ROS

## Abstract

**Objective:**

*Cuscuta campestris* or common dodder is a holoparasitic plant that has been valorized for treatment of liver injury and cancer prevention in traditional medicine. Recently, extract of *C. campestris* had shown moderate antimicrobial properties and cytotoxic effects. In this study, we examined the level of cellular oxidants, cytotoxicity, apoptosis and differentiation induced by hydroalcoholic extract of *C. campestris* (CCE) (12.5-200 µg/ml), as well as arsenic trioxide (As_2_O_3_, 50 µM), in human leukemic (HL60 and NB4) and normal polymorph nuclear cells after 72 hr treatment.

**Materials and Methods:**

Resazurin assay was used to determine cell viability following treatment with *C. campestris*. Intracellular reactive oxygen species (ROS) and apoptotic cells were measured by fluorimetry using carboxy 2′, 7′-dichlorofluorescein diacetate and propidium iodide (PI), as staining reagents, respectively. The differentiation of leukemic cells was evaluated by Giemsa staining and nitro blue tetrazolium (NBT) reduction.

**Results:**

*C. campestris* inhibited cell viability with IC_50_ values of 23.9 µg/ml for HL60 and 60.3 µg/ml for NB4 cells after 72 hr treatment. ROS formation was also concentration-dependently increased following treatment with *C. campestris*. In addition, the number of apoptotic cells significantly increased to 88.4% and 62.3% in CCE (200 µg/ml)-treated HL60 and NB4 cells, respectively, which was higher than that of As_2_O_3_ (50 µM)-treated leukemic cells (p<0.001). Nonetheless, *C. campestris* did not induce differentiation of leukemic cells towards granulocytic pattern.

**Conclusion:**

The present study demonstrated that *C. campestris* induced apoptosis through ROS production without having differential effect on leukemic cells, in concentration- and time-dependent manners. Understanding of precise signaling pathway by which *C. campestris* induce apoptosis, needs further research.

## Introduction

As hematologic malignancy, acute promyelocytic leukemia (APL) exhibits myeloid differentiation failure (Rodak et al., 2013 ▶). Arsenic trioxide (As_2_O_3_) is used clinically for treatment of APL; its anti-cancer mechanism includes induction of apoptosis, inhibition of growth, and promotion of differentiation. High doses of As_2_O_3 _cause various side effects and toxicity; however, substitution or combination of this drug with other anti-cancer compounds can reduce the required dose and side effects of As_2_O_3_ (Rodak et al., 2013 ▶). Therefore, search for novel anticancer agents is currently receiving great attention. Researchers believe that dietary phytochemicals may influence chemotherapy treatment and help to cure cancer (Alinejad et al., 2013 ▶). Different natural compounds can improve the efficiency of chemotherapeutic agents, decrease the resistance to these drugs, and alleviate their adverse effects (Sak, 2012 ▶). As a result, researchers attempt to employ different medicinal herbs and their effective phytochemicals in *in-vitro* and *in-vivo* models of cancer. 

*Cuscuta* spp., also known as dodder is one of the medicinal herbs that belongs to the Convolvulaceae family and there are over 150 species of dodders in the world (McNeal et al., 2007 ▶). *C. campestris* or common dodder is a holoparasitic plant that been valorized for treatment of liver injury and cancer prevention in traditional medicine. *C. campestris* has mild laxative and diuretic properties and can be used for treatment of sciatica, scurvy and scrofula derma (Holm, 1997 ▶). Pharmacological properties of this herb include anti-seizure, anti-microbial and cytotoxic effects against cancer cell lines. The active compounds of *Cuscuta* species include flavonoids, lignans, quinic acid and polysaccharides (Mehrabani et al., 2007 ▶, Biswas et al., 2012 ▶). 

In the present study, we investigated the cytotoxic and differentiation effects of *C. campestris* on human leukemic (HL60 and NB4) cells, compared to As_2_O_3_ (as positive control).

## Materials and Methods

**Preparation of the hydroalcoholic extract of** ***C. campestris*** **(CCE)**

The aerial parts of *C. campestris* were prepared in the early autumn 2015 from Razavi Khorasan province (the Northeast of Iran) and authenticated by the herbarium of Ferdowsi University of Mashhad (No. 38195 FUMH). The aerial parts of the plant were cleaned and grounded to fine powder using a blender. Then, macerated extract was prepared as previously described (Shafiee-Nick et al., 2012 ▶). Briefly, 150 g of the powder was suspended in 500 ml of 56% ethanol and incubated for 72 hr at 37°C. The hydro-alcoholic extract was then dried on a water bath and the residue was dissolved in distilled water (The yield of extract was 14%).

**Cell lines and reagents**

HL60 and NB4 cells were obtained from the cell bank of Pasteur Institute (Tehran, Iran). Resazurin reagent [300 μM resazurin, 78 μM methylene blue, 1 mM potassium hexacyanoferrate III and 1 mM potassium hexacyanoferrate II], 2,7-dichlorofluorescin diacetate (DCFH-DA), propidium iodide (PI), nitroblue tetrazolium (NBT), phorbol-myristate-acetate (PMA) and arsenic trioxide (stock concentration 1 mM) were purchased from Sigma (St Louis, MO). High-glucose Roswell Park Memorial Institute medium (RPMI1640), penicillin-streptomycin and fetal bovine serum (FBS) were obtained from Gibco BRL Life Technologies (Grand Island, NY). Giemsa stain was purchased from MERK (Darmstadt, Germany).

**Human normal cells isolation and culture**

Polymorphonuclear cells (PMNs) were obtained from healthy volunteers under sterile conditions by two consecutive Ficoll-Hypaque (Pharmacia, Country) density gradient centrifugations (Cassatella et al., 1988 ▶). PMNs were purified to more than 97%, as judged by morphological examination of Wright-stained smears. After washing with sterile phosphate-buffered saline (PBS), the cells were resuspended in RPMI medium. HL60, NB4 and PMN cells were maintained in RPMI medium supplemented with 10% (v/v) fetal bovine serum, 100 units/ml penicillin, and 100 µg/ml streptomycin at 37^◦^ C in a humidified atmosphere (90%) containing 5% CO_2_. Next, the cells were incubated with various concentrations of *C. campestris* extract (12.5-200 µg/ml) and As_2_O_3_ (50 µM) for 24, 48 and 72 hr. For each concentration and time course, there was a control sample which remained untreated and received an equal volume of medium. All different treatments were carried out in triplicate.

**Cell viability**

The cell viability was determined by resazurin reagent. For this purpose, 100 µl of the NB4, HL60 and PMN cells containing 1×10^5^ cells were added to each well in 96-well tissue culture plates, then cells were treated with *C. campestris* extract (12.5-200 µg/ml) and As_2_O_3_ (50 µM) for 24, 48 and 72 hr. Then, 20 µl of resazurin reagent was added to each well and the plates were incubated for 4 hr. The fluorescence intensity of the product resorufin which is proportional to the number of viable cells per well, was measured by a fluorescence Victor X5 2030 Multilabel Plate Reader (PerkinElmer, Shelton, Connecticut) with excitation at 530 nm and emission at 590 nm (Mashkani et al., 2016 ▶). 

**Measurement of reactive oxygen species**

In brief, HL60 and NB4 cells (10^5^/well) were incubated with DCF-DA reagent (20 µM) for 30 min in the dark and then treated with *C. campestris* extract (12.5-200 µg/ml) or As_2_O_3_ (50 µM) for 2 hr. After that, the cells were transferred to a Falcon polystyrene tube. After washing twice with PBS, The DCF fluorescence intensity was detected using a fluorescence Victor X5 2030 Multilabel Plate Reader (PerkinElmer, Shelton, Connecticut) with excitation wavelength set at 485 nm and emission wavelength set at 530 nm (Hosseini and Rajabian, 2016 ▶).

**Apoptosis**

Apoptotic cells were detected using PI staining of the treated cells followed by flow cytometry to detect the so-called sub-G1 peak. Briefly, HL60 and NB4 cells were treated with *C. campestris* extract (50-200 µg/ml) or As_2_O_3_ (50 µM) for 48 hr. The cells were then incubated at 4 °C overnight in the dark with 500 µl of a PI hypotonic buffer (50 µg/ml). Samples were then analyzed by flow cytometry. A total of 10,000 events per sample were obtained and the data was analyzed using WinMDI software (Sadeghnia et al., 2014 ▶).

**Morphological and differentiation assay**

Morphological characterization and differentiation assay were done by standard techniques using Giemsa-stained smears and was performed NBT reduction tests, respectively. Cells (1 × 10^6^ cells/ml in 6-well plates) were treated with *C. campestris* extract (12.5 and 25 µg/ml) or As_2_O_3_ (0.5 µM) (Yan et al., 2005 ▶) for 3 days and then, washed twice with PBS and the pellets were resuspended in equal volumes of 0.2% NBT dissolved in Dulbecco’s phosphate-buffered saline containing 200 ng/ml of freshly diluted phorbol myristate acetate (PMA). After 25 min incubation at 37°C in the dark, slides were prepared and stained with Giemsa and 300 cells were scored for the presence of blue-black formazan granules (Gupta et al., 2016 ▶). 

**Statistical analysis**

Statistical analysis were done by Graph Pad PRISM software (version 6, Graph Pad Software, Inc., San Diego, CA) using one-way analysis of variance (ANOVA) and Tukey’s multiple comparison post-tests to evaluate differences among several treatment groups. p-values less than 0.05 were considered as statistically significant. The values were presented as the mean ± SEM of three independent experiments.

## Results

***C. campestris*** **decreased cell viability in a concentration-dependent manner**

**Figure 1:**
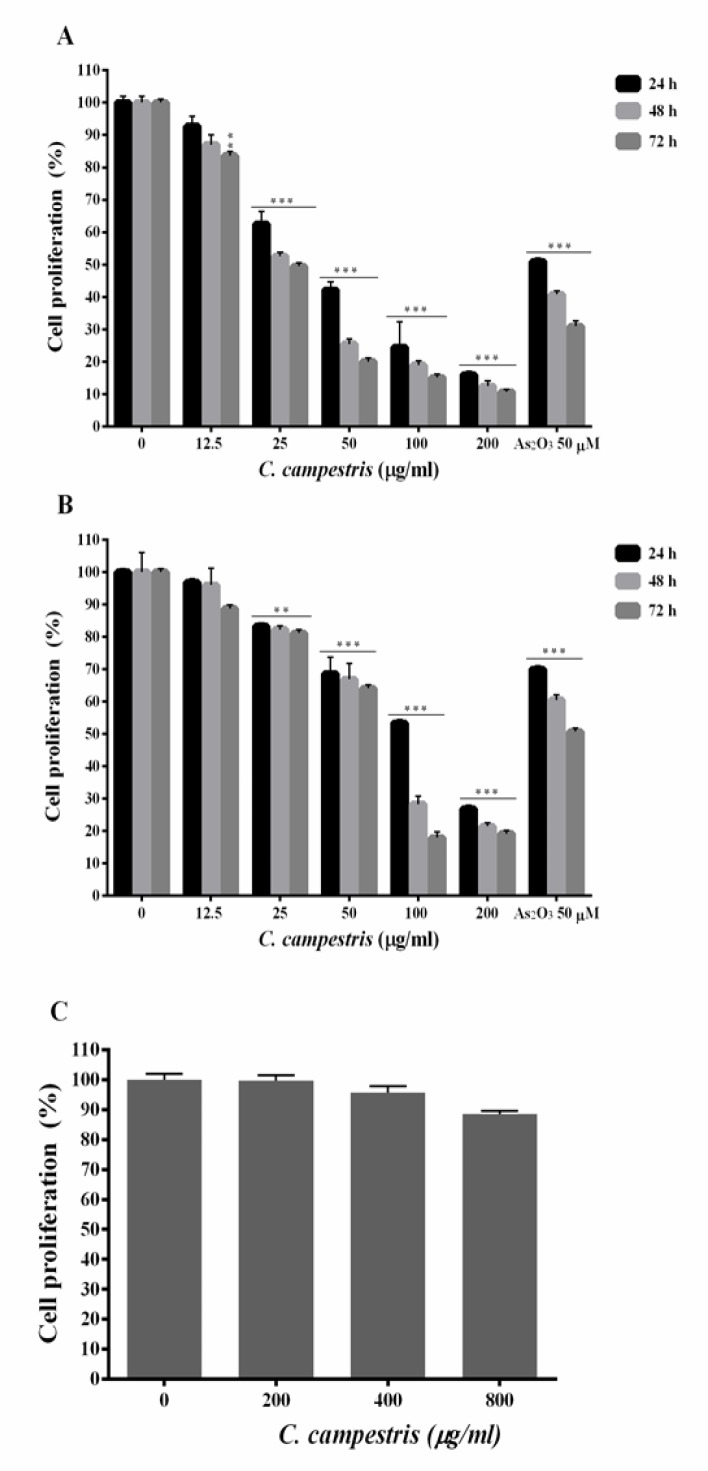
Anti-proliferative effect of *C. campestris* on leukemic cells. (A) HL60 and (B) NB4 cells were treated with different concentrations of *C. campestris* (12.5-200 µg/ml) or As_2_O_3_ (50 µM) for 24-72 hr. (C) Normal polymorphnuclear cells treated with *C. campestris* (200-800 µg/ml) for 24 hr. The percentage of cell viability (quantitated by resazurin assay) was normalized against the negative controls for each cell type. Data are expressed as the mean ± SEM of three separate experiments. **p<0.01 and ^***^p<0.001 as compared to control value

To evaluate the toxic effect of *C. campestris*, HL60 and NB4 cells were incubated with increasing concentrations of *C. campestris* (12.5-200 µg/ml) and As_2_O_3_ (50 µM), as a positive control, for up to 72 hr and the cell viability was measured by resazurin reagent (Figure 1). The normal PMN cells were incubated with *C. campestris* (200-800 µg/ml) for 24 hr. As compared to PMN cells, *C. campestris*-induced toxicity was significantly higher in the HL60 and NB4 cells. The results are graphically shown in Figure 1 and summarized in Table 1. Treatment of HL60 cells with relatively high concentration (>50 µg/ml) of *C. campestris *for 24, 48 and 72 hr significantly reduced cell viability to 42.1 ± 2.5, 25.4 ± 1.6 and 20.0 ± 1.0%, at 100 µg/ml, 24.4 ± 7.9, 18.9 ± 1.2 and 15.1 ± 1.0%, at 200 µg/ml, and 16.0 ± 1.0, 12.4 ± 1.6 and 10.7 ± 0.6% against 50.9 ± 1.0, 40.8 ± 1.0 and 30.9 ± 1.6% in As_2_O_3_-treated cells (p<0.001), respectively. While in NB4 cells, *C. campestris *at concentrations >100 µg/ml resulted in more significant reductions in cell viability compared to As_2_O_3_, at 100 µg/ml 53.3±1.0, 28.3±2.4 and 17.9±1.7%, at 200 µg/ml 26.8±1.1, 21.4±1.2 and 19.2±1.1% against 70.0±1.0, 60.5±1.6 and 50.7±1.0% in As_2_O_3_ group (p<0.001). In contrast, the extract did not exhibit any cytotoxic effects, at concentrations up to 800 µg/ml, in PMN cells after 24 hr treatment (Figure. 1).

***C. campestris*** **concentration-dependently increased ROS formation**

**Figure 2:**
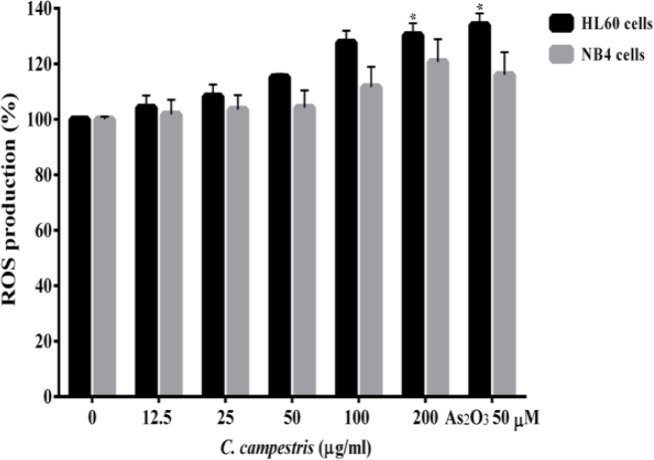
Effect of *C. campestris *on intracellular reactive oxygen species (ROS) in leukemic cells. NB4 and HL60 cells were treated with different concentrations of *C. campestris *(12.5-200 µg/ml) or As_2_O_3_ (50 µM) for 2 hr. Data are expressed as the mean ± SEM of three separate experiments. ^*^p<0.05 as compared to control value

Our findings showed a regular and statistically significant time and concentration-dependent increase in ROS generation in HL60 and NB4 cells, 2 hr after treatment with *C. campestris *compared to control group (Figure 2). As compared with control HL60 cells, 200 µg/ml *C. campestris *significantly increased (p<0.001) ROS formation (130 ± 4.1%, comparable to that of As_2_O_3_ group; 134 ± 3.5%). There was also a significant increase (p<0.001) in ROS level in NB4 cells 2 hr after treatment with 200 µg/ml of *C. campestris *compared to control group (120 ± 8.1% comparable to that of As_2_O_3_ group; 116 ± 7.1%).

***C. campestris*** **concentration-dependently induced apoptotic cell death **

**Figure 3:**
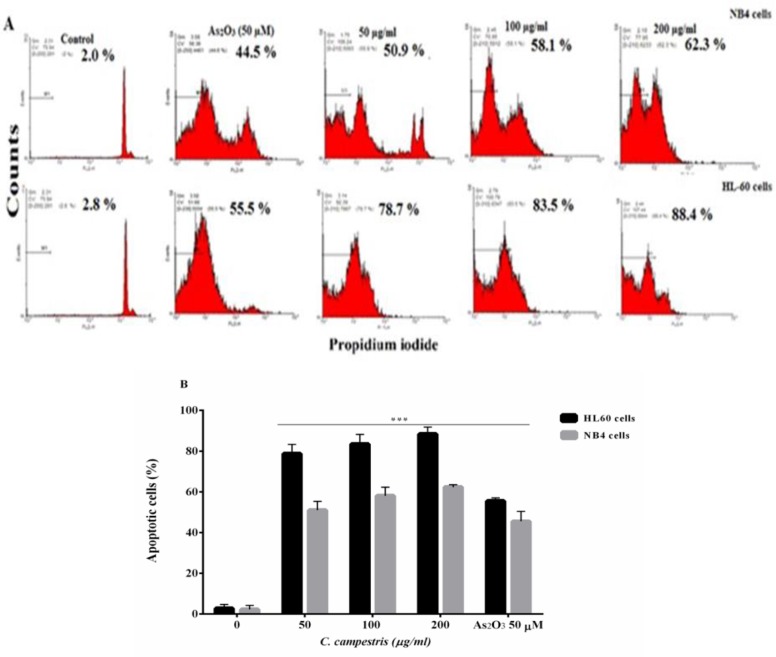
Apoptotic cell death induced by *C. campestris *in leukemic cells. (A) NB4 and HL60 cells were incubated with different concentrations of *C. campestris *(12.5-200 µg/ml) or As_2_O_3_ (50 µM) for 48 hr. Apoptosis was assayed by PI staining and analyzed by flow cytometry. (B) Apoptosis rate shown as bars. The data shown are the means ± SEM of three independent experiments. ^***^p<0.001 as compared to control value

Figure 3 shows that *C. campestris* at high concentration induces apoptosis in NB4 and HL60 cells, significantly. After 48 hr of treatment, *C. campestris*-treated HL60 cells showed 83.5 ± 4.7% (100 μg/ml) and 88.4 ± 3.4% (200 μg/ml) apoptosis rate, comparable to As_2_O_3_ (50 µM)-treated cells (55.5±1.5%) (p<0.001). Also, 62.3 ± 1.1% of *C. campestris*-treated NB4 cells undergone apoptosis at 200 μg/ml as compared to 45.5 ± 4.9% in As_2_O_3_ group (p<0.001). These results showed that the number of apoptotic cells increased with increasing concentrations, as compared to control cells (p<0.001, Figure 3). 

***C. campestris*** **did not induce differentiation of leukemic cells into granulocyte pattern**

**Figure 4:**
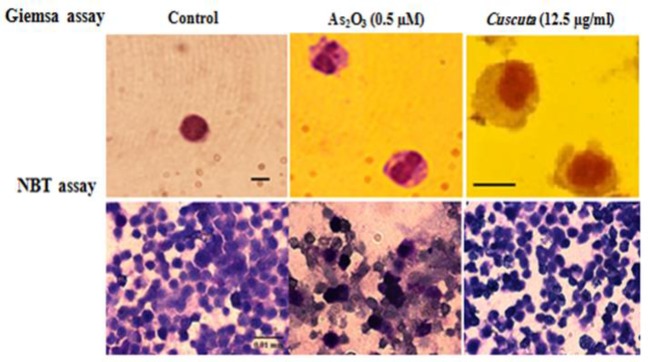
Effect of *C. campestris *on morphological and functional properties of granulocyte in leukemic cells. NB4 cells were treated with *C. campestris *(12.5 µM) or As_2_O_3_ (0.5 µM) for 72 hr. Morphologic and functional properties of granulocytes were determined by Giemsa and NBT assays, respectively. In Giemsa-stained slides, As_2_O_3_-treated cells (as positive control) showed polymorphonuclear morphology of granulocyte and *C. campestris*-treated cells showed promyelocytes characteristics similar to control. On NBT slides, As_2_O_3_-treated cells (as positive control) showed intracellular blue-black formazan deposits while *C. campestris*-treated cells showed no intracellular blue-black formazan deposits (bar represents 0.01 mm

The Giemsa and NBT assays showed that *C. campestris *did not influence the maturation of leukemic cells toward neutrophils. In morphological Giemsa assay, cells showed promyelocytes characteristics with cytoplasmic granules, similar to control cells. In functional NBT assay, cells had no intracellular blue-black formazan deposits (Figure. 4).

**Table 1.** IC_50_ (concentration required for 50% inhibition) values of different treatments of *C. campestris* against NB4 and HL60 cells at 24-72 hr

**Cell lines**	**HL60 cells**			**NB4 cells**		
**Treatment**	**24 hr**	**48 hr**	**72 hr**	**24 hr**	**48 hr**	**72 hr**
***C. campestris*** ** (µg/ml)**	43.09±0.05	29.10±0.06	24.85±0.04	105.7±0.03	73.63±0.06	60.36±05

## Discussion

This study is the first to investigate the antineoplastic and differentiating effects of *C. campestris *in leukemic cells. We reported that *C. campestris *exhibits significant toxicity in the cancer cells, at tested concentrations. The cytotoxic effects of *C. campestris *were more pronounced against the neoplastic cells compared to human PMN cells. Our study also showed that *C. campestris *induces concentration-dependent apoptosis by increasing ROS generation. It is noteworthy to mention that these effects are comparable to standard anti-leukemic drug, As_2_O_3_. The results also showed that the rate of apoptosis induced by *C. campestris* in HL-60 (AML M2) cells was higher than that of NB4 (AML M3) cells. These may be related to inherent metabolic properties of the cancer cells. Previous studies indicated that NB4 cells show highly ‘‘glycolytic’’ properties (Suganuma et al., 2010 ▶, Estañ et al., 2014 ▶). Recent studies have also shown that some species of *Cuscuta *such as *C. reflexa* exhibit anti-tumor properties and are used for the treatment of prostate cancer (Suresh et al., 2011 ▶). It was shown that *C. epithyum* significantly reduced the viability of Hela, HT-29 and MDA-MB-468 cells (Jafarian et al., 2014 ▶). Previous studies also assessed the effects of different species of *Cuscuta* on various cell lines including lymphoblastic-like tumor cell line (HL60), epidermoid carcinoma cell line (MCF-7), human breast cancer cell line (T47D), human Caucasian acute lymphoblastic leukemia (CCRF-CEM) and Jurkat (JM) cell lines (Sepehr et al., 2011 ▶, Ghazanfari et al., 2013 ▶). 

The phytochemical studies have shown that active compounds of *Cuscuta* species are flavonoids, lignans, quinic acid and polysaccharides. Flavonoids are considered effective antioxidants (Sepehr et al., 2011 ▶). Also, several studies have demonstrated that the pharmacological effects of *C. chinensis* and *C. epithymum* can be attributed to their main constituents including flavonoids, saccharids, alkaloids, lignans, saponins, and resin glycosides. Also, it has been shown that many flavonoids significantly decreased cell viability via enhancement of caspase activity (Sepehr et al., 2011 ▶). In the same way, several steroidal saponins possess marked cancer chemopreventive properties (Candra et al., 2002 ▶, Kajimoto et al., 2002 ▶, Yokosuka et al., 2002 ▶, Choi et al., 2003 ▶, Hu and Yao, 2003 ▶, Shen et al., 2003 ▶). The triterpene saponins, jenisseensosides A-D were found to increase the accumulation and cytotoxicity of the anticancer agent, cisplatin in human colon tumor cells (Gaidi et al., 2002 ▶). 

In conclusion, the present study demonstrated that *C. campestris *inhibited the proliferation without affecting the differentiation of leukemic cells. The marked difference in cytotoxicity between cancer and normal cells suggests that *C. campestris *could be considered as a novel alternative to cancer therapeutics. The IC_50_ values reported in our study may become considerably lower if the isolated active compounds are used. *C. campestris *not only increased levels of ROS, but also induced concomitant increase of apoptosis. The precise signaling pathway via which *C. campestris *induce apoptosis needs further research. 
